# Protocol for a systematic review of self-management interventions for older adults living with cancer

**DOI:** 10.1186/s13643-020-01346-1

**Published:** 2020-04-17

**Authors:** Kristen R. Haase, Martine Puts, Schroder Sattar, Mikaela Gray, Cindy Kenis, Valentina Donison, Steven Hall, Bianca McLean, Aria Wills, Doris Howell

**Affiliations:** 1 College of Nursing, University of Saskatchewan, 4th Floor, Health Sciences E-Wing, 104 Clinic Place, Saskatoon, Saskatchewan S7N 2Z4 Canada; 2 Lawrence S. Bloomberg Faculty of Nursing, University of Toronto, Toronto, ON Canada; 3 College of Nursing, University of Saskatchewan, Regina, Canada; 4 Gerstein Science Information Centre, University of Toronto, Toronto, Canada; 5 Department of General Medical Oncology and Geriatric Medicine, University Hospitals Leuven, Leuven, Belgium; 6 Daphne Cockwell School of Nursing, Ryerson University, Toronto, ON Canada; 7 De Groote School of Medicine, McMaster University, Hamilton, ON Canada; 8 Princess Margaret Hospital, University of Toronto, Toronto, ON Canada

**Keywords:** Self-management, Cancer, Older adults, Systematic review

## Abstract

**Background:**

Cancer predominates in adults over age 65. Cancer treatments are known to create physical and psychosocial challenges, which may be amplified for older adults with cancer.

Learning and applying self-management behaviours and skills during treatment with cancer can help to manage/recover health and improve quality of life. In many other chronic illnesses, self-management interventions are known to improve health outcomes and lower healthcare costs. The purpose of this systematic review is to determine the effectiveness of self-management interventions for older adults with cancer on physical, psychosocial, and health system-related outcomes.

**Methods:**

We are conducting a systematic review of self-management interventions for older adults (65+) diagnosed with cancer (solid tumour or haematological) in the active treatment phase of cancer. This systematic review is guided by the Preferred Reporting Items for Systematic Reviews and Meta-analysis (PRISMA) statement. Studies are limited to experimental or quasi-experimental methods published in English, French, German, or Dutch. A search strategy was designed with a Health Sciences librarian and performed using the following electronic databases: Ageline, AMED, ASSIA, Cinahl, Cochrane, Embase, Medline, PsychINFO, and Sociological Abstracts. Approximately 14,000 titles and abstracts are being electronically screened by a minimum of 2 reviewers, with relevant studies to be screened for full text. The final sample of included studies will be assessed for quality using the Cochrane Risk of Bias tool and Down and Black for quasi-experimental studies, with data synthesized in a narrative and tabular format.

**Discussion:**

This systematic review will expand the knowledge base of interventions supporting self-management for older adults with cancer. This study will inform future intervention development by identifying gaps and strengths in effective self-management interventions targeting the needs of older adults receiving active treatment for cancer.

**Systematic review registration:**

PROPERO registry ID# CRD42019134113

## Background

Cancer is the most common disease in Canada and worldwide [1]. In Canada, 60% of all new cancer cases occur in those over age 70 and cancer is the leading cause of death for adults in this age group [2]. While the number of older adults in Canada is expected to double in the next 20 years, a corresponding 40% increase in new cancer cases is expected, the majority of which will occur among older adults [3]. Cancer is a complex illness, and ultimately, patients and their families bear the responsibility for managing the negative and distressing physical and psychosocial effects of cancer and cancer treatments [4, 5]. For patients and their families to meet the challenges accompanying cancer, they must mobilize knowledge and supports to coordinate treatment, manage their illness, and navigate the complexities of the healthcare system [6].

Self-management is defined as the ability to manage the disease and treatment effects and psychosocial changes arising as a result of illness [7] and comprises several core skills and tasks. Lorig and Hollman [8] indicate that effective management of a chronic illness requires patients to be supported with respect to three key tasks (medical, emotional, and role management), and six skills (problem-solving, decision-making, resource use, partnering with providers, action planning, and self-tailoring). These core self-management skills enable people to manage their disease and treatment effects, thereby reducing impact on functioning in daily life and optimizing health. Despite the important role that self-management can play for people with cancer, patients report seldom receiving support to engage in illness self-management and clinicians may lack the requisite knowledge or time to support patients’ skills and application of self-management behaviours [9]. When patients lack self-management skills, they cannot anticipate potential treatment side effects or engage in proactive behaviours, which would enable them to avoid costly, stressful, and time-consuming emergency room visits. Indeed, many older adults lack self-management support and monitoring skills, leaving them vulnerable to greater morbidity, mortality, disability, functional decline, and exacerbation of their comorbid chronic diseases [9]. Extensive reviews of evidence related to other chronic non-malignant diseases demonstrate improved clinical disease parameters (e.g. reduction in A1C in diabetes, improvements post-stroke) and lower costs when patients are active in self-management [10–13]; however, less is known about the effectiveness and components of self-management interventions for older adults with cancer.

## Objective

The purpose of this systematic review is to determine the effectiveness and components of existing self-management interventions for older adults (> 65 years) with cancer on physical, psychosocial, and health system-related outcomes in the acute phase of cancer.

Our specific research questions are as follows:

1. What is the impact of self-management interventions on patient outcomes?
2. What are the components of effective self-management interventions?
3. Are there differences in effectiveness depending on participant characteristics? For example, regarding age, tumour type (solid tumours and haematological malignancies), and stage?
4. What are the strengths and gaps in effective self-management interventions for older adults with cancer?

## Methods and design

To achieve this objective, we have chosen a systematic review approach, which according to the Cochrane Collaboration defines as a research method which ‘summarises the results of available carefully designed healthcare studies (controlled trials) and provides a high level of evidence on the effectiveness of healthcare interventions.’ [14]. Our interdisciplinary team is composed of experts in cancer nursing (KH, MP, SS, DH), self-management (DH), epidemiology (MP, DH), geriatrics (MP, SS, CK), a health sciences librarian (MG), and a team of interdisciplinary trainees (VD, SH, BM, AW), and is well positioned to execute this review.

### Study design

This systematic review is guided by the Preferred Reporting Items for Systematic Reviews and Meta-Analyses (PRISMA) statement [15], and the PRISMA-P guidelines were used to structure this protocol (Additional file 1) [16]. This systematic review is registered in the Prospective Register of Systematic Reviews (PROSPERO) database (registration number: CRD42019134113). As advised in the PRISMA statement, we use the PICOH acronym (population, intervention, comparison, outcomes, health care context) [17], to systematically address our research question and plan our search strategy.

#### Population

Our population of focus is older adults (≥ 65 years) diagnosed with cancer of any tumour type (solid tumours and haematological malignancies) at stages 1–3. We are excluding interventions targeting those with stage 4 cancer or advanced cancer, as interventions targeting stage 4 cancer with an older adult population may be more geared towards palliation and end of life strategies [18]. Generating knowledge regarding older adults with cancer is important, as ageing is identified as the most important risk factor for cancer [19], and there is limited (but growing) evidence supporting the care of older adults with cancer [20]. We will include studies with a mean study population age of 65 years or above or include a subgroup analysis of these individuals. In our review, we will include studies of persons with cancer undergoing active treatment, those who choose no treatment, or those who have completed treatment.

#### Interventions

The interventions of interest will focus on enabling older adults to self-manage the effects of cancer during the diagnosis or active treatment phase of cancer. These interventions may be community-based or patient-focused interventions and include singular or multiple components related to self-management, as defined in the relevant literature [7, 21], including problem-solving, decision-making, symptom management, social support, resource utilization, communication, education and/or information sharing, formation of a patient-provider partnership, action planning, or self-tailoring. Because we are including older adults with cancer undergoing active treatment, those who choose no treatment, or those who have completed treatment, some of these interventions may be defined as ‘survivorship’ interventions. This is further complicated by the broadest definition of the term survivorship, which is defined as starting at the time of diagnosis [22, 23]. Therefore, we will include interventions labelled as survivorship interventions and screen them further to determine whether they fit the study inclusion criteria.

#### Comparators

The self-management intervention defined above will be compared to any of the following: usual patient education, waiting list control, no treatment, written information only, or another active intervention that is not considered to be self-management.

#### Outcomes

Our primary outcomes of interest relevant to self-management interventions include patient behaviours, self-efficacy, quality of life, symptom management/burden, well-being, and adjustment or psychological distress. Secondary outcomes of interest include program usability and health care utilization or costs. These outcomes will be included in the current review within the context of interventions for older adults with cancer.

#### Healthcare context

The context of interest to this study is community-based, outpatient, or ambulatory cancer settings. Interventions delivered in the home or community will be included.

### Eligibility criteria

In addition to the above requirements, studies meeting the following *inclusion* criteria will be included in the review:

- Mean age of the study population was 65 years or over or if mean/median age was < 65 but reported subgroup analysis of older adults with a mean/median age ≥ 65
- Published in English, Dutch, French, or German
- Study designs: RCTs and quasi-experimental designs (with or without control)
- No date limit

Studies will be *excluded* based on the following criteria:

- Editorials, case studies, reviews, expert opinion papers, and studies that were published as abstracts only

### Information sources

#### Search strategy

Searches were conducted in Ovid MEDLINE (1946 to present, including Epub Ahead of Print and In-Process & Other Non-Indexed Citations), Ovid Embase (1947 to present), Ovid AMED (1985 to present), OVID PsycINFO (1806 to present), EBSCO CINAHL Plus with Full Text (1981 to present), EBSCO AgeLine, Cochrane CENTRAL, ProQuest ASSIA, and ProQuest Sociological Abstracts to identify articles addressing cancer self-management interventions for and older adults with cancer. Search strategies were developed by an academic health science librarian (MG) in collaboration with the project team. The Medline search strategy was peer-reviewed by a second academic health science librarian not associated with the project; recommendations made by the reviewer were addressed by the team and incorporated prior to translating the search to additional databases. The search strategies were executed and translated using platform-specific syntax for each database, controlled vocabulary, and appropriate search fields. MeSH terms, Emtree terms, CINAHL headings, APA thesaurus terms, AMED thesaurus terms, and textwords were used to search the concepts of self-management, cancer, and older adults. No date, language, or study design limits were placed on the search. See Appendix 1 for Medline search strategies.

#### Study records

##### Data management and selection process

Citations for the studies located through the aforementioned search strategy were imported into EndNote X9 Referencing Software. We used a step-wise process for de-duplication following the method described by Bramer [24], using both automatic and manual strategies to locate and remove duplicates. The library of citations was then uploaded into Covidence systematic review software [25].

Titles and abstracts of studies retrieved using the aforementioned search strategy will be screened independently by a team of reviewers, with a minimum of two people screening each potential study to meet the inclusion criteria outlined above. We will use Covidence to organize, manage, and monitor the screening and selection process. All studies will be screened by the first (KH), second (MP), or third author (SS). Conflicts will be resolved by a PhD prepared team member, not involved in the prior stages of screening (CK). The full-text articles of potentially eligible studies will be retrieved and independently assessed for eligibility by team members. All full-text papers will be screened by the first (KH), second (MP), or third author (SS). In instances where multiple articles reported similar results and are deemed to be from the same data set, the most comprehensive article will be retained. We will also review reference lists to search for additional articles for forward referencing. Any disagreement over the eligibility of particular studies will be resolved through discussion with the study team.

#### Data collection process

##### Data abstraction

 We will abstract information from each selected study regarding general information (e.g. title, registration status, authors), intervention (e.g. intervention name, description, type, theoretical basis length of intervention, and delivery context), study characteristics (e.g. study aim(s), design, sampling method, recruitment procedures (inclusion/exclusion criteria), sample size for each group, data sources, recruitment type, and participant characteristics: sex, tumour type (solid tumours and haematological malignancies), and stage; treatment type; comorbidities), and outcomes (e.g. measures, change scores, effect size). For all articles with missing details, corresponding authors were contacted via email to request the information. All data were independently extracted using a standardized Microsoft Excel sheet by two authors.

Risk of bias in individual studies will be assessed using two tools, depending on the methods of the respective studies. For randomized clinical trials, we will use the Cochrane Risk of Bias tool (revised 2019 version) [26]. For quasi-experimental studies, we will use Downs and Black’s checklist for assessing methodological quality of non-randomized studies [27]. We will use the quality assessment tools to inform conclusions about the content and nature of the body of work.

### Data synthesis and analysis

We will present the PRISMA flow diagram to demonstrate the process of study selection, inclusion, and exclusion [28]. Extracted and synthesized data will be presented in both narrative and tabular format. The narrative synthesis will provide detailed descriptions of the included studies, with an interest in the parameters outlined by Popay and colleagues [29] including (1) examining how and for whom the intervention works. For example, given the diversity of cancers, we are interested in tumour type (solid tumours and haematological malignancies), stage, and treatment variation; (2) synthesizing the included studies including the direction and size of effects; and (3) exploring relationships in the data. A summary of key data points, including study characteristics, intervention details, and risk of bias assessment, will also be presented in accompanying tables.

Based on our knowledge of the self-management literature, we anticipate heterogeneity amongst the intervention types, components, and outcomes, which will limit pooled analysis. We will also endeavour to evaluate differences in effectiveness related to intervention type, timing of the intervention (both in terms of duration and at which stage in the cancer trajectory the intervention is initiated), and tumour type (solid tumours and haematological malignancies). The risk of bias assessment will provide context for the study findings and support the confidence in the evaluation of the state of the science. Studies determined to be of poor quality will be retained to ensure we have a comprehensive understanding of the range of interventions.

We will use the GRADE (Grading of Recommendations, Assessment, Development and Evaluations) [30] approach to rate the evidence and make a determination of the effectiveness of self-management interventions for older adults with cancer.

## Discussion

Screening for this study is ongoing. Assessing the effectiveness of self-management interventions and their effective components for older adults with cancer will add to the knowledge base in geriatric oncology. We anticipate that the results of this systematic review will direct future research and expand the knowledge base regarding self-management interventions for older adults with cancer. This study will inform future research efforts by identifying gaps and strengths in effective self-management interventions targeting the needs of older adults receiving active treatment for cancer.

Our knowledge translation strategy includes presenting at a Canadian dissemination meeting, publishing peer-reviewed outlets, and conference presentations. The dissemination meeting will convene a group of Canadian cancer stakeholders, including patients, clinicians, researchers, and administrators, to establish research priorities for self-management in older adults with cancer. The lead author (KH) will also share these study findings with an advisory group of older adults with cancer to inform future research directions.

### Supplementary information

**Additional file 1.** PRISMA-P 2015 Checklist

## Appendix

**Table 1:** All searches run May 8, 2019. Ovid MEDLINE: Epub Ahead of Print, In-Process & Other Non-Indexed Citations, Ovid MEDLINE® Daily and Ovid MEDLINE® <1946-Present>

#	Searches	Results
1	exp Neoplasms/	3163602
2	(neoplas* or paraneoplas* or cancer* or tumor or tumour or oncolog* or metast* or malignan*).tw,kf.	2903078
3	1 or 2 [cancer]	4001856
4	self care/	31505
5	self administration/	10889
6	self medication/	4550
7	Self-Help Groups/	8804
8	Self-Control/	1735
9	diagnostic self evaluation/	2766
10	self-assessment/	12034
11	self efficacy/	18401
12	Self-Management/	1102
13	(psychoeducation* or psycho-education*).tw,kf.	5668
14	((self or oneself or herself or himself or themsel*) adj3 (administ* or aid or assert* or assess* or assur* or care* or caring or control* or depend* or determin* or diagnos* or direct* or dosag* or dose* or dosing or evaluat* or efficac* or efficienc* or exam* or govern* or guide* or guidance or help* or maintain* or manag* or medicat* or monitor* or motivat* or regulat* or refer* or rely* or relian* or serve* or serving or service* or subsist* or sufficien* or sustain* or support* or triag*)).tw,kf.	232893
15	or/4-14 [self-management]	280877
16	exp Aged/	2936291
17	Geriatrics/	29175
18	Aging/	220157
19	Health Services for the Aged/	17113
20	(ageing or aging or aged or elder* or geriatric* or old age* or senior* or older).tw,kf.	1191141
21	or/16-20 [older adults]	3714266
22	3 and 15 and 21	9051

## Abbreviations

PICOH: Patient, intervention, comparison, outcomes, healthcare context
PRISMA-P: Preferred Reporting Items for Systematic Review and Meta-analysis Protocols
